# 11-(4-Meth­oxy­phen­yl)-3,3-dimethyl-2,3,4,5,10,11-hexa­hydro-1*H*-dibenzo[*b*,*e*][1,4]diazepin-1-one monohydrate

**DOI:** 10.1107/S1600536812016194

**Published:** 2012-04-25

**Authors:** Olatomide A. Fadare, Pius O. Adelani, Adebomi A. Ikotun, Craig A. Obafemi

**Affiliations:** a Chemistry Department, Obafemi Awolowo University, Ile-ife, Nigeria; b Department of Civil Engineering and Geological Sciences, and Department of Chemistry and Biochemistry, University of Notre Dame, Notre Dame, Indiana 46556, USA; c Chemistry and Industrial Chemistry Department, Bowen University, Iwo, Nigeria

## Abstract

In the title compound, C_22_H_24_N_2_O_2_·H_2_O, the co-crystallized water mol­ecule inter­acts with the N and O atoms of the mol­ecule through O_w_—H⋯N, O_w_—H⋯O(meth­yl) and N—H⋯O_w_ hydrogen-bonding inter­actions. These hydrogen bonds, along with the inter­molecular N—H⋯O=C hydrogen-bonding inter­actions, connect the mol­ecules into a three-dimensional network. The dihedral angle between the two aromatic rings is 65.46 (10)°.

## Related literature

For details of the synthesis, see: Hanze *et al.* (1963 ▶); Rashed *et al.* (1993 ▶); Kolos *et al.* (2004 ▶); Cortés *et al.* (2007 ▶); Ajani *et al.* (2010 ▶). For the biological activity of dibenzo[*b*,*e*][1,4]diazepinones, see: Beccalli *et al.* (2005 ▶); Farnet *et al.* (2005 ▶); Joergensen *et al.* (1996 ▶); McAlpine *et al.* (2008 ▶); McGowan *et al.* (2009 ▶).
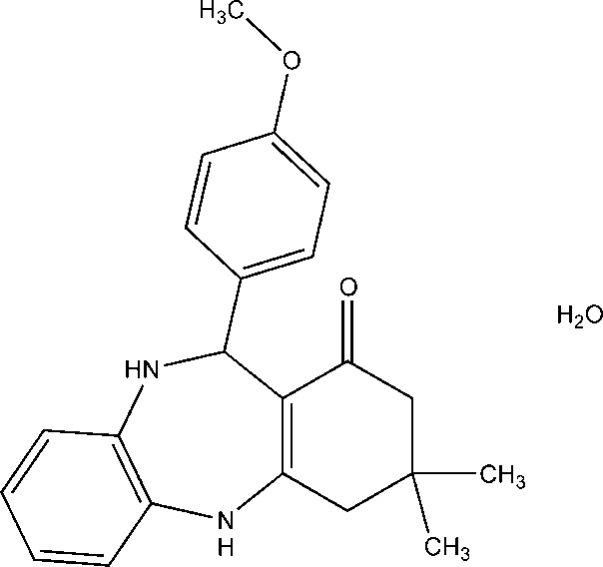


## Experimental

### 

#### Crystal data

C_22_H_24_N_2_O_2_·H_2_O
*M* *_r_* = 366.45
Monoclinic, 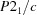

*a* = 10.684 (7) Å
*b* = 16.973 (12) Å
*c* = 11.174 (8) Å
β = 101.490 (9)°
*V* = 1986 (2) Å^3^
*Z* = 4
Mo *K*α radiation
μ = 0.08 mm^−1^
*T* = 296 K
0.04 × 0.02 × 0.01 mm

#### Data collection

Bruker APEXII CCD diffractometer
Absorption correction: multi-scan (*SADABS*; Bruker, 2004 ▶) *T*_min_ = 0.244, *T*_max_ = 0.323
22692 measured reflections
4529 independent reflections
2187 reflections with *I* > 2σ(*I*)
*R*_int_ = 0.087

#### Refinement

*R*[*F* ^2^ > 2σ(*F* ^2^)] = 0.043
*wR*(*F* ^2^) = 0.106
*S* = 0.83
4529 reflections
257 parameters
H atoms treated by a mixture of independent and constrained refinement
Δρ_max_ = 0.19 e Å^−3^
Δρ_min_ = −0.16 e Å^−3^

### 

Data collection: *APEX2* (Bruker, 2004 ▶); cell refinement: *SAINT* (Bruker, 2004 ▶); data reduction: *SAINT*; program(s) used to solve structure: *SHELXS97* (Sheldrick, 2008 ▶); program(s) used to refine structure: *SHELXL97* (Sheldrick, 2008 ▶); molecular graphics: *SHELXTL* (Sheldrick, 2008 ▶); software used to prepare material for publication: *SHELXTL*.

## Supplementary Material

Crystal structure: contains datablock(s) I, global. DOI: 10.1107/S1600536812016194/ff2060sup1.cif

Structure factors: contains datablock(s) I. DOI: 10.1107/S1600536812016194/ff2060Isup2.hkl

Supplementary material file. DOI: 10.1107/S1600536812016194/ff2060Isup3.cml

Additional supplementary materials:  crystallographic information; 3D view; checkCIF report

## Figures and Tables

**Table 1:** Hydrogen-bond geometry (Å, °)

*D*—H⋯*A*	*D*—H	H⋯*A*	*D*⋯*A*	*D*—H⋯*A*
N1—H1*A*⋯O1*W*^i^	0.86	2.14	2.994 (3)	170
O1*W*—H1*B*⋯N2^ii^	0.98 (3)	2.04 (3)	3.020 (3)	178.2 (14)
O1*W*—H1*C*⋯O2	0.99 (2)	1.85 (2)	2.832 (3)	170 (2)
N2—H2*C*⋯O1^iii^	0.949 (18)	2.099 (18)	3.047 (3)	177.4 (13)

Symmetry codes: (i) 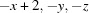; (ii) 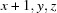; (iii) 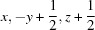.

**Fig. 1.**
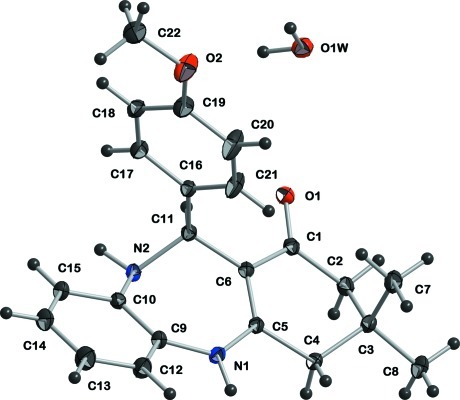
The molecular structure of the title compound showing the labelled atoms; thermal ellipsoids are drawn at the 20% probability level.

**Fig. 2.**
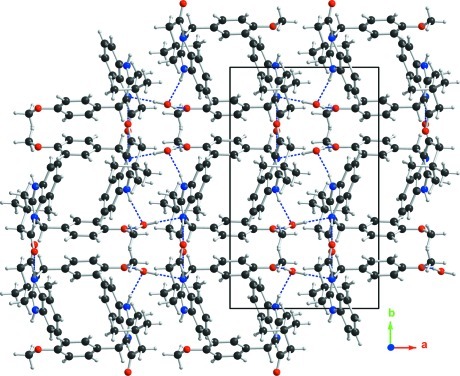
The packing diagram (ball and stick model) of the title compound, viewed along c-direction. Hydrogen bonds are drawn as dashed lines.

**Fig. 3.**
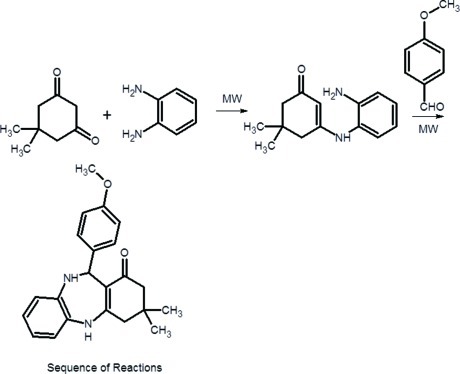
Reaction scheme of synthesis of the title compound.

## supplementary crystallographic information

### Comment

Dibenzo[b,e][1,4]diazepinones are useful intermediates in the synthesis of pharmaceuticals. For example, dibenzo[b,e][1,4]diazepin-11-ones are useful intermediates in the preparation of dibenzo[1,4]diazepines (Hanze *et al.*, 1963). They display wide variety of biological properties, including antidepressant (Beccalli *et al.*, 2005), antimicrobial (Farnet *et al.*, 2005), analgesic and anti-inflammatory (Joergensen *et al.*, 1996), antitumor (McAlpine *et al.*, 2008) activities, while the dibenzodiazepin-1-ones are hepatitis C virus (HCV) NS5B polymerase inhibitors (McGowan *et al.*, 2009). In view of our interest in bioactivity of nitrogen-containing heterocyclic compounds (see: Ajani *et al.*, 2010), we report here the microwave-assisted synthesis and the crystal structure of the title compound 1.

### Experimental

A mixture of dimedone (2.0 g, 14.3 mmol) and *o*-phenylenediamine (1.54 g, 14.3 mmol) in absolute ethanol (30 ml), containing acetic acid (0.5 ml), in a beaker was pulse irradiated in a microwave oven for 5 min. and left to stand at room temperature for 1 h. Anisaldehyde (1.95 g, 14.3 mmol) was added to the reaction mixture and then subjected to microwave irradiation for 5 min. (TLC monitored). Evaporation of the solvent afforded a gummy product. Cold aqueous ethanol was added with scratching to give yellow precipitate (melting point 202–205 ^o^ C). Crystals of 1 suitable for X-ray analysis were obtained by slow evaporation of ethanol solution of the product.

### Refinement

The H atoms of the water molecule were located on a Fourier difference map, restrained by *DFIX* command 0.85 for O—H distances and by *DFIX* 1.39 for H···H distance, and refined as riding with *U*_iso_(H) = 1.5Ueq(O). Other atoms were placed in their calculated positions, with C—H = 0.93 or 0.96 Å, and refined using a riding model, with *U*_iso_(H) = 1.2 or 1.5Ueq(C).

### Crystal data

**Table:**

C_22_H_24_N_2_O_2_·H_2_O	*F*(000) = 784
*M**_r_* = 366.45	*D*_x_ = 1.226 Mg m^−^^3^
Monoclinic, *P*2_1_/*c*	Mo *K*α radiation, λ = 0.71073 Å
Hall symbol: -P 2ybc	Cell parameters from 2566 reflections
*a* = 10.684 (7) Å	θ = 2.2–21.4°
*b* = 16.973 (12) Å	µ = 0.08 mm^−^^1^
*c* = 11.174 (8) Å	*T* = 296 K
β = 101.490 (9)°	Rectangular tablet, light yellow
*V* = 1986 (2) Å^3^	0.04 × 0.02 × 0.01 mm
*Z* = 4	

### Data collection

**Table:**

Bruker APEXII CCD diffractometer	4529 independent reflections
Radiation source: fine-focus sealed tube	2187 reflections with *I* > 2σ(*I*)
Graphite monochromator	*R*_int_ = 0.087
φ and ω scans	θ_max_ = 27.5°, θ_min_ = 1.9°
Absorption correction: multi-scan (*SADABS*; Bruker, 2004)	*h* = −13→13
*T*_min_ = 0.244, *T*_max_ = 0.323	*k* = −21→22
22692 measured reflections	*l* = −14→14

### Refinement

**Table:**

Refinement on *F*^2^	Secondary atom site location: difference Fourier map
Least-squares matrix: full	Hydrogen site location: inferred from neighbouring sites
*R*[*F*^2^ > 2σ(*F*^2^)] = 0.043	H atoms treated by a mixture of independent and constrained refinement
*wR*(*F*^2^) = 0.106	*w* = 1/[σ^2^(*F*_o_^2^) + (0.0442*P*)^2^] where *P* = (*F*_o_^2^ + 2*F*_c_^2^)/3
*S* = 0.83	(Δ/σ)_max_ < 0.001
4529 reflections	Δρ_max_ = 0.19 e Å^−^^3^
257 parameters	Δρ_min_ = −0.16 e Å^−^^3^
0 restraints	Extinction correction: *SHELXL97* (Sheldrick, 2008), Fc^*^=kFc[1+0.001xFc^2^λ^3^/sin(2θ)]^-1/4^
Primary atom site location: structure-invariant direct methods	Extinction coefficient: 0.0082 (9)

### Special details

**Table:**

Geometry. All e.s.d.'s (except the e.s.d. in the dihedral angle between two l.s. planes) are estimated using the full covariance matrix. The cell e.s.d.'s are taken into account individually in the estimation of e.s.d.'s in distances, angles and torsion angles; correlations between e.s.d.'s in cell parameters are only used when they are defined by crystal symmetry. An approximate (isotropic) treatment of cell e.s.d.'s is used for estimating e.s.d.'s involving l.s. planes.
Refinement. Refinement of *F*^2^ against ALL reflections. The weighted *R*-factor *wR* and goodness of fit *S* are based on *F*^2^, conventional *R*-factors *R* are based on *F*, with *F* set to zero for negative *F*^2^. The threshold expression of *F*^2^ > σ(*F*^2^) is used only for calculating *R*-factors(gt) *etc*. and is not relevant to the choice of reflections for refinement. *R*-factors based on *F*^2^ are statistically about twice as large as those based on *F*, and *R*- factors based on ALL data will be even larger.

### Fractional atomic coordinates and isotropic or equivalent isotropic displacement parameters (Å^2^)

**Table:**

	*x*	*y*	*z*	*U*_iso_*/*U*_eq_	
N1	0.69308 (13)	−0.00644 (8)	0.04879 (12)	0.0381 (4)	
H1A	0.6703	−0.0509	0.0142	0.046*	
N2	0.68575 (13)	0.12181 (9)	0.21811 (13)	0.0341 (4)	
H2C	0.6841 (17)	0.1561 (10)	0.2848 (17)	0.053 (6)*	
O1	0.68552 (12)	0.26436 (7)	−0.07109 (11)	0.0459 (4)	
O2	1.28931 (13)	0.17001 (10)	0.27104 (13)	0.0787 (5)	
O1W	1.41964 (16)	0.15384 (8)	0.07513 (13)	0.0520 (4)	
H1C	1.371 (2)	0.1659 (14)	0.140 (2)	0.104 (9)*	
H1B	1.506 (3)	0.1427 (15)	0.120 (2)	0.124 (11)*	
C1	0.66212 (16)	0.19349 (11)	−0.09321 (15)	0.0346 (4)	
C2	0.59266 (18)	0.16858 (11)	−0.21814 (15)	0.0425 (5)	
H2A	0.6057	0.2081	−0.2772	0.051*	
H2B	0.5019	0.1661	−0.2186	0.051*	
C3	0.63682 (17)	0.08909 (11)	−0.25676 (15)	0.0401 (5)	
C4	0.61823 (18)	0.03053 (10)	−0.15837 (15)	0.0398 (5)	
H4A	0.5275	0.0210	−0.1658	0.048*	
H4B	0.6577	−0.0190	−0.1732	0.048*	
C5	0.67258 (15)	0.05645 (10)	−0.02950 (15)	0.0318 (4)	
C6	0.69591 (15)	0.13400 (10)	0.00061 (15)	0.0307 (4)	
C7	0.77701 (19)	0.09265 (13)	−0.26960 (18)	0.0574 (6)	
H7A	0.8292	0.1086	−0.1933	0.086*	
H7B	0.7859	0.1300	−0.3318	0.086*	
H7C	0.8036	0.0416	−0.2917	0.086*	
C8	0.5557 (2)	0.06348 (13)	−0.37901 (17)	0.0596 (6)	
H8A	0.4676	0.0610	−0.3724	0.089*	
H8B	0.5832	0.0125	−0.4005	0.089*	
H8C	0.5655	0.1009	−0.4410	0.089*	
C9	0.74429 (16)	−0.01265 (10)	0.17528 (15)	0.0361 (4)	
C10	0.74023 (16)	0.04797 (10)	0.25838 (15)	0.0349 (4)	
C11	0.74741 (16)	0.16327 (10)	0.12753 (15)	0.0322 (4)	
H11A	0.7198	0.2183	0.1283	0.039*	
C12	0.79555 (19)	−0.08505 (11)	0.21730 (17)	0.0506 (5)	
H12A	0.7985	−0.1254	0.1617	0.061*	
C13	0.8421 (2)	−0.09834 (13)	0.33978 (19)	0.0606 (6)	
H13A	0.8757	−0.1473	0.3666	0.073*	
C14	0.8386 (2)	−0.03852 (13)	0.42190 (18)	0.0567 (6)	
H14A	0.8694	−0.0469	0.5048	0.068*	
C15	0.78916 (18)	0.03402 (11)	0.38124 (16)	0.0451 (5)	
H15A	0.7886	0.0744	0.4374	0.054*	
C16	0.89251 (16)	0.16503 (10)	0.16580 (15)	0.0319 (4)	
C17	0.94955 (17)	0.19550 (11)	0.27789 (16)	0.0423 (5)	
H17A	0.8978	0.2149	0.3290	0.051*	
C18	1.08113 (18)	0.19834 (11)	0.31741 (17)	0.0439 (5)	
H18A	1.1167	0.2191	0.3936	0.053*	
C19	1.15759 (18)	0.17016 (13)	0.24243 (18)	0.0508 (5)	
C20	1.1030 (2)	0.14011 (16)	0.1304 (2)	0.0809 (9)	
H20A	1.1548	0.1213	0.0791	0.097*	
C21	0.97209 (19)	0.13747 (14)	0.09301 (18)	0.0625 (7)	
H21A	0.9370	0.1166	0.0168	0.075*	
C22	1.35131 (19)	0.19213 (14)	0.38866 (18)	0.0643 (7)	
H22A	1.4421	0.1892	0.3946	0.096*	
H22B	1.3265	0.1572	0.4474	0.096*	
H22C	1.3278	0.2451	0.4046	0.096*	

### Atomic displacement parameters (Å^2^)

**Table:**

	*U*^11^	*U*^22^	*U*^33^	*U*^12^	*U*^13^	*U*^23^
N1	0.0504 (10)	0.0309 (9)	0.0314 (9)	−0.0015 (7)	0.0045 (7)	−0.0035 (7)
N2	0.0364 (9)	0.0381 (9)	0.0299 (8)	−0.0002 (7)	0.0113 (7)	−0.0040 (7)
O1	0.0576 (9)	0.0375 (8)	0.0420 (8)	−0.0038 (7)	0.0082 (6)	0.0037 (6)
O2	0.0309 (8)	0.1414 (15)	0.0598 (11)	0.0007 (9)	−0.0007 (7)	−0.0285 (10)
O1W	0.0470 (9)	0.0548 (9)	0.0536 (10)	−0.0013 (7)	0.0083 (8)	−0.0027 (7)
C1	0.0310 (10)	0.0394 (11)	0.0342 (11)	−0.0007 (8)	0.0083 (8)	0.0025 (9)
C2	0.0436 (12)	0.0475 (12)	0.0343 (11)	−0.0036 (9)	0.0031 (9)	0.0068 (9)
C3	0.0414 (11)	0.0503 (12)	0.0273 (10)	−0.0052 (9)	0.0034 (8)	−0.0031 (9)
C4	0.0459 (11)	0.0398 (11)	0.0315 (10)	−0.0048 (9)	0.0026 (8)	−0.0033 (8)
C5	0.0302 (10)	0.0372 (11)	0.0277 (10)	−0.0007 (8)	0.0050 (8)	0.0011 (8)
C6	0.0273 (9)	0.0363 (11)	0.0285 (10)	0.0012 (8)	0.0055 (7)	0.0005 (8)
C7	0.0547 (14)	0.0787 (16)	0.0417 (12)	−0.0058 (12)	0.0164 (10)	−0.0086 (11)
C8	0.0718 (15)	0.0698 (15)	0.0315 (11)	−0.0090 (12)	−0.0035 (10)	−0.0008 (10)
C9	0.0406 (11)	0.0384 (11)	0.0289 (10)	−0.0023 (9)	0.0057 (8)	0.0017 (8)
C10	0.0337 (10)	0.0414 (11)	0.0301 (10)	−0.0037 (8)	0.0075 (8)	0.0003 (9)
C11	0.0325 (10)	0.0330 (10)	0.0317 (10)	0.0009 (8)	0.0076 (8)	−0.0014 (8)
C12	0.0680 (15)	0.0400 (12)	0.0413 (12)	0.0052 (10)	0.0053 (10)	0.0018 (10)
C13	0.0780 (17)	0.0504 (14)	0.0476 (14)	0.0080 (12)	−0.0010 (12)	0.0123 (11)
C14	0.0714 (16)	0.0598 (15)	0.0342 (12)	−0.0066 (12)	−0.0011 (10)	0.0102 (11)
C15	0.0541 (13)	0.0500 (13)	0.0298 (11)	−0.0085 (10)	0.0052 (9)	−0.0001 (9)
C16	0.0310 (10)	0.0342 (10)	0.0300 (10)	0.0004 (8)	0.0049 (8)	−0.0010 (8)
C17	0.0358 (11)	0.0522 (12)	0.0390 (12)	−0.0019 (9)	0.0077 (9)	−0.0100 (9)
C18	0.0419 (12)	0.0506 (12)	0.0367 (11)	−0.0049 (10)	0.0019 (9)	−0.0064 (9)
C19	0.0284 (11)	0.0751 (15)	0.0464 (13)	−0.0006 (10)	0.0017 (9)	−0.0082 (11)
C20	0.0339 (13)	0.153 (3)	0.0556 (15)	0.0073 (14)	0.0094 (11)	−0.0419 (15)
C21	0.0357 (12)	0.1076 (19)	0.0418 (13)	0.0026 (12)	0.0020 (10)	−0.0293 (12)
C22	0.0390 (13)	0.0911 (18)	0.0553 (15)	−0.0085 (12)	−0.0084 (11)	0.0041 (13)

### Geometric parameters (Å, º)

**Table:**

N1—C5	1.370 (2)	C8—H8B	0.9600
N1—C9	1.414 (2)	C8—H8C	0.9600
N1—H1A	0.8600	C9—C12	1.389 (2)
N2—C10	1.417 (2)	C9—C10	1.392 (2)
N2—C11	1.490 (2)	C10—C15	1.388 (2)
N2—H2C	0.949 (19)	C11—C16	1.524 (2)
O1—C1	1.243 (2)	C11—H11A	0.9800
O2—C19	1.380 (2)	C12—C13	1.378 (3)
O2—C22	1.400 (2)	C12—H12A	0.9300
O1W—H1C	1.00 (3)	C13—C14	1.374 (3)
O1W—H1B	0.97 (3)	C13—H13A	0.9300
C1—C6	1.449 (2)	C14—C15	1.380 (3)
C1—C2	1.505 (2)	C14—H14A	0.9300
C2—C3	1.520 (3)	C15—H15A	0.9300
C2—H2A	0.9700	C16—C21	1.371 (2)
C2—H2B	0.9700	C16—C17	1.380 (2)
C3—C4	1.524 (2)	C17—C18	1.388 (3)
C3—C8	1.528 (2)	C17—H17A	0.9300
C3—C7	1.534 (3)	C18—C19	1.368 (3)
C4—C5	1.507 (2)	C18—H18A	0.9300
C4—H4A	0.9700	C19—C20	1.369 (3)
C4—H4B	0.9700	C20—C21	1.378 (3)
C5—C6	1.369 (2)	C20—H20A	0.9300
C6—C11	1.500 (2)	C21—H21A	0.9300
C7—H7A	0.9600	C22—H22A	0.9600
C7—H7B	0.9600	C22—H22B	0.9600
C7—H7C	0.9600	C22—H22C	0.9600
C8—H8A	0.9600		
			
C5—N1—C9	132.62 (15)	C12—C9—N1	116.87 (16)
C5—N1—H1A	113.7	C10—C9—N1	123.65 (16)
C9—N1—H1A	113.7	C15—C10—C9	118.53 (17)
C10—N2—C11	115.11 (14)	C15—C10—N2	120.98 (16)
C10—N2—H2C	111.4 (11)	C9—C10—N2	120.48 (16)
C11—N2—H2C	108.9 (11)	N2—C11—C6	110.93 (14)
C19—O2—C22	119.15 (16)	N2—C11—C16	112.42 (14)
H1C—O1W—H1B	104 (2)	C6—C11—C16	115.65 (14)
O1—C1—C6	121.29 (16)	N2—C11—H11A	105.7
O1—C1—C2	119.90 (16)	C6—C11—H11A	105.7
C6—C1—C2	118.76 (16)	C16—C11—H11A	105.7
C1—C2—C3	112.89 (15)	C13—C12—C9	121.37 (19)
C1—C2—H2A	109.0	C13—C12—H12A	119.3
C3—C2—H2A	109.0	C9—C12—H12A	119.3
C1—C2—H2B	109.0	C14—C13—C12	119.3 (2)
C3—C2—H2B	109.0	C14—C13—H13A	120.3
H2A—C2—H2B	107.8	C12—C13—H13A	120.3
C2—C3—C4	106.36 (15)	C13—C14—C15	119.93 (19)
C2—C3—C8	110.71 (15)	C13—C14—H14A	120.0
C4—C3—C8	109.11 (16)	C15—C14—H14A	120.0
C2—C3—C7	110.83 (15)	C14—C15—C10	121.43 (18)
C4—C3—C7	111.29 (16)	C14—C15—H15A	119.3
C8—C3—C7	108.53 (16)	C10—C15—H15A	119.3
C5—C4—C3	114.71 (15)	C21—C16—C17	116.91 (17)
C5—C4—H4A	108.6	C21—C16—C11	122.92 (16)
C3—C4—H4A	108.6	C17—C16—C11	120.17 (15)
C5—C4—H4B	108.6	C16—C17—C18	122.55 (17)
C3—C4—H4B	108.6	C16—C17—H17A	118.7
H4A—C4—H4B	107.6	C18—C17—H17A	118.7
C6—C5—N1	126.45 (16)	C19—C18—C17	118.93 (18)
C6—C5—C4	122.15 (15)	C19—C18—H18A	120.5
N1—C5—C4	111.40 (15)	C17—C18—H18A	120.5
C5—C6—C1	119.04 (16)	C18—C19—C20	119.49 (19)
C5—C6—C11	124.43 (15)	C18—C19—O2	124.40 (18)
C1—C6—C11	116.42 (16)	C20—C19—O2	116.12 (18)
C3—C7—H7A	109.5	C19—C20—C21	120.7 (2)
C3—C7—H7B	109.5	C19—C20—H20A	119.6
H7A—C7—H7B	109.5	C21—C20—H20A	119.6
C3—C7—H7C	109.5	C16—C21—C20	121.38 (19)
H7A—C7—H7C	109.5	C16—C21—H21A	119.3
H7B—C7—H7C	109.5	C20—C21—H21A	119.3
C3—C8—H8A	109.5	O2—C22—H22A	109.5
C3—C8—H8B	109.5	O2—C22—H22B	109.5
H8A—C8—H8B	109.5	H22A—C22—H22B	109.5
C3—C8—H8C	109.5	O2—C22—H22C	109.5
H8A—C8—H8C	109.5	H22A—C22—H22C	109.5
H8B—C8—H8C	109.5	H22B—C22—H22C	109.5
C12—C9—C10	119.41 (17)		

### Hydrogen-bond geometry (Å, º)

**Table:**

*D*—H···*A*	*D*—H	H···*A*	*D*···*A*	*D*—H···*A*
N1—H1*A*···O1*W*^i^	0.86	2.14	2.994 (3)	170
O1*W*—H1*B*···N2^ii^	0.98 (3)	2.04 (3)	3.020 (3)	178.2 (14)
O1*W*—H1*C*···O2	0.99 (2)	1.85 (2)	2.832 (3)	170 (2)
N2—H2*C*···O1^iii^	0.949 (18)	2.099 (18)	3.047 (3)	177.4 (13)

Symmetry codes: (i) −*x*+2, −*y*, −*z*; (ii) *x*+1, *y*, *z*; (iii) *x*, −*y*+1/2, *z*+1/2.
